# Supplementary material for: eDNA reveals spatial differences in species composition of protected rockfishes
Source: PLoS One. 2025 Dec 1;20(12):e0337724. doi: 10.1371/journal.pone.0337724 (PMC12668609; doi:10.1371/journal.pone.0337724)
Supplement: Attachment — Submitted filename: Response_to_Reviewers_09-18.docx [file pone.0337724.s005.docx]

Dear Dr. Fujiwara,

We thank yourself and two anonymous reviewers for the time taken to provide constructive suggestions to improve our manuscript, ‘eDNA reveals spatial differences in species composition of protected rockfish’. Please find below a point-by-point response to the suggested edits.

All the best,

Stephanie Matthews

Journal Requirements:

When submitting your revision, we need you to address these additional requirements.

1.Please ensure that your manuscript meets PLOS ONE's style requirements, including those for file naming. The PLOS ONE style templates can be found at

[https://journals.plos.org/plosone/s/file?id=wjVg/PLOSOne_formatting_sample_main_body.pdf](https://urldefense.com/v3/__https:/journals.plos.org/plosone/s/file?id=wjVg*PLOSOne_formatting_sample_main_body.pdf__;Lw!!K-Hz7m0Vt54!nQb11Tk8pmTbbMs8aJ-9W1RfmYAuoFVbbTqlyheztpgXiTiHwcKcCaqP3uVsuFyN_ampfDycG3Qiwg$) and

[https://journals.plos.org/plosone/s/file?id=ba62/PLOSOne_formatting_sample_title_authors_affiliations.pdf](https://urldefense.com/v3/__https:/track.editorialmanager.com/CL0/https:*2F*2Fjournals.plos.org*2Fplosone*2Fs*2Ffile*3Fid=ba62*2FPLOSOne_formatting_sample_title_authors_affiliations.pdf/1/010f01987a003173-05bdeb4f-4a57-4e3d-bd39-e25c90f80a65-000000/mZm9gH6dl-wmK6iZKpXLMbacKZOEyXZh6uLUZwticVk=222__;JSUlJSUlJQ!!K-Hz7m0Vt54!nQb11Tk8pmTbbMs8aJ-9W1RfmYAuoFVbbTqlyheztpgXiTiHwcKcCaqP3uVsuFyN_ampfDwf_k3R9w$)

We have updated our file formatting, including the formatting of the body of the text, references, figures, and author information.

2. In your Methods section, please provide additional information regarding the permits you obtained for the work. Please ensure you have included the full name of the authority that approved the field site access and, if no permits were required, a brief statement explaining why.

We have moved the statement explaining why permits were not required to a stand-alone paragraph for clarity.

3. Please note that funding information should not appear in any section or other areas of your manuscript. We will only publish funding information present in the Funding Statement section of the online submission form. Please remove any funding-related text from the manuscript.

All funding-related text has been removed from the manuscript file.

4. Thank you for stating the following financial disclosure:

“This project was funded in part by the National Philanthropic Trust.”

Please state what role the funders took in the study.  If the funders had no role, please state: "The funders had no role in study design, data collection and analysis, decision to publish, or preparation of the manuscript."

If this statement is not correct you must amend it as needed.

Please include this amended Role of Funder statement in your cover letter; we will change the online submission form on your behalf.

We have added the amended description of the Role of Funder to our cover letter. It should read:

“This project was funded in part by the National Philanthropic Trust. The funders had no role in study design, data collection and analysis, decision to publish, or preparation of the manuscript."

5. When completing the data availability statement of the submission form, you indicated that you will make your data available on acceptance. We strongly recommend all authors decide on a data sharing plan before acceptance, as the process can be lengthy and hold up publication timelines. Please note that, though access restrictions are acceptable now, your entire data will need to be made freely accessible if your manuscript is accepted for publication. This policy applies to all data except where public deposition would breach compliance with the protocol approved by your research ethics board. If you are unable to adhere to our open data policy, please kindly revise your statement to explain your reasoning and we will seek the editor's input on an exemption. Please be assured that, once you have provided your new statement, the assessment of your exemption will not hold up the peer review process.

All data have been uploaded to NCBI and are freely available, or will be released upon acceptance.

6. We note that Figure 2 in your submission contain map/satellite images which may be copyrighted. All PLOS content is published under the Creative Commons Attribution License (CC BY 4.0), which means that the manuscript, images, and Supporting Information files will be freely available online, and any third party is permitted to access, download, copy, distribute, and use these materials in any way, even commercially, with proper attribution. For these reasons, we cannot publish previously copyrighted maps or satellite images created using proprietary data, such as Google software (Google Maps, Street View, and Earth). For more information, see our copyright guidelines: [http://journals.plos.org/plosone/s/licenses-and-copyright](https://urldefense.com/v3/__http:/journals.plos.org/plosone/s/licenses-and-copyright__;!!K-Hz7m0Vt54!nQb11Tk8pmTbbMs8aJ-9W1RfmYAuoFVbbTqlyheztpgXiTiHwcKcCaqP3uVsuFyN_ampfDwRx16qHQ$).

 We require you to either (1) present written permission from the copyright holder to publish these figures specifically under the CC BY 4.0 license, or (2) remove the figures from your submission:

 1. You may seek permission from the original copyright holder of Figure 2 to publish the content specifically under the CC BY 4.0 license.

 We recommend that you contact the original copyright holder with the Content Permission Form ([http://journals.plos.org/plosone/s/file?id=7c09/content-permission-form.pdf](https://urldefense.com/v3/__http:/journals.plos.org/plosone/s/file?id=7c09*content-permission-form.pdf__;Lw!!K-Hz7m0Vt54!nQb11Tk8pmTbbMs8aJ-9W1RfmYAuoFVbbTqlyheztpgXiTiHwcKcCaqP3uVsuFyN_ampfDz3IHVnaQ$)) and the following text:

“I request permission for the open-access journal PLOS ONE to publish XXX under the Creative Commons Attribution License (CCAL) CC BY 4.0 ([http://creativecommons.org/licenses/by/4.0/](https://urldefense.com/v3/__http:/creativecommons.org/licenses/by/4.0/__;!!K-Hz7m0Vt54!nQb11Tk8pmTbbMs8aJ-9W1RfmYAuoFVbbTqlyheztpgXiTiHwcKcCaqP3uVsuFyN_ampfDyWTwLiIw$)). Please be aware that this license allows unrestricted use and distribution, even commercially, by third parties. Please reply and provide explicit written permission to publish XXX under a CC BY license and complete the attached form.”

 Please upload the completed Content Permission Form or other proof of granted permissions as an "Other" file with your submission.

 In the figure caption of the copyrighted figure, please include the following text: “Reprinted from [ref] under a CC BY license, with permission from [name of publisher], original copyright [original copyright year].”

 2. If you are unable to obtain permission from the original copyright holder to publish these figures under the CC BY 4.0 license or if the copyright holder’s requirements are incompatible with the CC BY 4.0 license, please either i) remove the figure or ii) supply a replacement figure that complies with the CC BY 4.0 license. Please check copyright information on all replacement figures and update the figure caption with source information. If applicable, please specify in the figure caption text when a figure is similar but not identical to the original image and is therefore for illustrative purposes only.

The following resources for replacing copyrighted map figures may be helpful:

 USGS National Map Viewer (public domain): [http://viewer.nationalmap.gov/viewer/](https://urldefense.com/v3/__http:/viewer.nationalmap.gov/viewer/__;!!K-Hz7m0Vt54!nQb11Tk8pmTbbMs8aJ-9W1RfmYAuoFVbbTqlyheztpgXiTiHwcKcCaqP3uVsuFyN_ampfDxLkHLVzQ$)

The Gateway to Astronaut Photography of Earth (public domain): [http://eol.jsc.nasa.gov/sseop/clickmap/](https://urldefense.com/v3/__http:/eol.jsc.nasa.gov/sseop/clickmap/__;!!K-Hz7m0Vt54!nQb11Tk8pmTbbMs8aJ-9W1RfmYAuoFVbbTqlyheztpgXiTiHwcKcCaqP3uVsuFyN_ampfDyPm2H-Yg$)

Maps at the CIA (public domain): [https://www.cia.gov/library/publications/the-world-factbook/index.html](https://urldefense.com/v3/__https:/www.cia.gov/library/publications/the-world-factbook/index.html__;!!K-Hz7m0Vt54!nQb11Tk8pmTbbMs8aJ-9W1RfmYAuoFVbbTqlyheztpgXiTiHwcKcCaqP3uVsuFyN_ampfDy6nczuhA$) and [https://www.cia.gov/library/publications/cia-maps-publications/index.html](https://urldefense.com/v3/__https:/www.cia.gov/library/publications/cia-maps-publications/index.html__;!!K-Hz7m0Vt54!nQb11Tk8pmTbbMs8aJ-9W1RfmYAuoFVbbTqlyheztpgXiTiHwcKcCaqP3uVsuFyN_ampfDyJAVQndw$)

NASA Earth Observatory (public domain): [http://earthobservatory.nasa.gov/](https://urldefense.com/v3/__http:/earthobservatory.nasa.gov/__;!!K-Hz7m0Vt54!nQb11Tk8pmTbbMs8aJ-9W1RfmYAuoFVbbTqlyheztpgXiTiHwcKcCaqP3uVsuFyN_ampfDwhrbk-Rg$)

Landsat: [http://landsat.visibleearth.nasa.gov/](https://urldefense.com/v3/__http:/landsat.visibleearth.nasa.gov/__;!!K-Hz7m0Vt54!nQb11Tk8pmTbbMs8aJ-9W1RfmYAuoFVbbTqlyheztpgXiTiHwcKcCaqP3uVsuFyN_ampfDyX7vcD7A$)

USGS EROS (Earth Resources Observatory and Science (EROS) Center) (public domain): [http://eros.usgs.gov/#](https://urldefense.com/v3/__http:/eros.usgs.gov/*__;Iw!!K-Hz7m0Vt54!nQb11Tk8pmTbbMs8aJ-9W1RfmYAuoFVbbTqlyheztpgXiTiHwcKcCaqP3uVsuFyN_ampfDwolaLQNQ$)

Natural Earth (public domain): [http://www.naturalearthdata.com/](https://urldefense.com/v3/__http:/track.editorialmanager.com/CL0/http:*2F*2Fwww.naturalearthdata.com*2F/1/010f01987a003173-05bdeb4f-4a57-4e3d-bd39-e25c90f80a65-000000/SZhtojeddKPEtJdBI3YzSo9IW5b3SzeijX_MqfvV9gk=222__;JSUl!!K-Hz7m0Vt54!nQb11Tk8pmTbbMs8aJ-9W1RfmYAuoFVbbTqlyheztpgXiTiHwcKcCaqP3uVsuFyN_ampfDwR9Xh5DQ$)

Figure 1 contains data from NOAA NCEI, which is not subject to copyright protection in the United States (https://data.noaa.gov/metaview/page?xml=NOAA/NESDIS/NGDC/MGG/DEM//iso/xml/etopo_2022.xml&view=getDataView&header=none).

We have added a statement to the figure caption including the data source and the statement that it is not subject to copyright restrictions. We have also updated the methods to include the R package used to create the map.

7. Please include captions for your Supporting Information files at the end of your manuscript, and update any in-text citations to match accordingly. Please see our Supporting Information guidelines for more information: [http://journals.plos.org/plosone/s/supporting-information](https://urldefense.com/v3/__http:/journals.plos.org/plosone/s/supporting-information__;!!K-Hz7m0Vt54!nQb11Tk8pmTbbMs8aJ-9W1RfmYAuoFVbbTqlyheztpgXiTiHwcKcCaqP3uVsuFyN_ampfDx86kSjpw$).

We have added labels and abbreviated captions for all supporting information to the end of the manuscript file.

8. If the reviewer comments include a recommendation to cite specific previously published works, please review and evaluate these publications to determine whether they are relevant and should be cited. There is no requirement to cite these works unless the editor has indicated otherwise.

We have reviewed the reviewer comments and provided a line-by-line response below.

Additional Editor Comments:

First, I would like to apologize for the delay in the review process. Initially, two reviewers agreed to evaluate the manuscript, but only one submitted their comments. We then had two additional reviewers, and again received comments from just one. Fortunately, we now have feedback from two reviewers who are well-versed in eDNA methods and their application to marine fish detection.

Both reviewers are generally positive about the manuscript and commend your efforts in tackling a technically challenging topic. However, they raised several important concerns that will need to be addressed. I am recommending a **Major Revision** to ensure the revised manuscript fully satisfies the reviewers' expectations.

**Key Points to Address:**

1. **Hybrid Detection and Mitochondrial Limitations**
   One reviewer noted that while hybridization is briefly mentioned, the manuscript does not sufficiently discuss its implications. Given the use of mitochondrial markers, which only reflect maternal lineages, I suggest including a paragraph in the Discussion that addresses:
   - The challenges of detecting hybrids with eDNA.
   - Potential biases in species richness estimates.
   - Relevant literature on this topic.
2. **Bioinformatics Transparency**
   There is concern about the lack of detail regarding bioinformatics methods. This is particularly important for a methodological journal like *PLOS ONE*. Please ensure all tools, parameters (e.g., trimming thresholds, software versions), and workflows are fully and clearly described.
3. **Mock Community Assay**
   One reviewer highlighted the absence of a mock community experiment as a limitation, especially regarding primer efficiency and species detectability. As this aspect falls outside my expertise, I will defer to the reviewers to evaluate your response to this point in the revised submission.

 In addition, there are several constructive suggestions related to figures, minor text edits, and data accessibility. Please incorporate these changes where appropriate. If you choose not to implement a particular suggestion, provide a brief rationale in your response to reviewers. The reviewers have contributed thoughtful and detailed feedback, and I am confident they would appreciate careful consideration of their comments.

The goal is to strengthen the manuscript so that it makes a greater impact within the scientific community.

[Note: HTML markup is below. Please do not edit.]

Reviewers' comments:

Reviewer's Responses to Questions

**Comments to the Author**

1. Is the manuscript technically sound, and do the data support the conclusions?

The manuscript must describe a technically sound piece of scientific research with data that supports the conclusions. Experiments must have been conducted rigorously, with appropriate controls, replication, and sample sizes. The conclusions must be drawn appropriately based on the data presented.

Reviewer #1: Yes

Reviewer #2: Yes

2. Has the statistical analysis been performed appropriately and rigorously?

Reviewer #1: Yes

Reviewer #2: N/A

3. Have the authors made all data underlying the findings in their manuscript fully available?

The [PLOS Data policy](https://urldefense.com/v3/__http:/track.editorialmanager.com/CL0/http:*2F*2Fwww.plosone.org*2Fstatic*2Fpolicies.action*23sharing/1/010f01987a003173-05bdeb4f-4a57-4e3d-bd39-e25c90f80a65-000000/VMFjR50RUk8Hhd5JK3qdNBPF-QjxxqHIRCJJTO5zCfo=222__;JSUlJSU!!K-Hz7m0Vt54!nQb11Tk8pmTbbMs8aJ-9W1RfmYAuoFVbbTqlyheztpgXiTiHwcKcCaqP3uVsuFyN_ampfDxLIqtyEA$) requires authors to make all data underlying the findings described in their manuscript fully available without restriction, with rare exception (please refer to the Data Availability Statement in the manuscript PDF file). The data should be provided as part of the manuscript or its supporting information, or deposited to a public repository. For example, in addition to summary statistics, the data points behind means, medians and variance measures should be available. If there are restrictions on publicly sharing data—e.g. participant privacy or use of data from a third party—those must be specified.

Reviewer #1: No

Reviewer #2: Yes

4. Is the manuscript presented in an intelligible fashion and written in standard English?

PLOS ONE does not copyedit accepted manuscripts, so the language in submitted articles must be clear, correct, and unambiguous. Any typographical or grammatical errors should be corrected at revision, so please note any specific errors here.

Reviewer #1: Yes

Reviewer #2: Yes

4. Review Comments to the Author

   Please use the space provided to explain your answers to the questions above. You may also include additional comments for the author, including concerns about dual publication, research ethics, or publication ethics. (Please upload your review as an attachment if it exceeds 20,000 characters)

Reviewer #1: This study demonstrates the power of eDNA, especially for a genus of fishes of commercial and ecological importance. It clearly contributes to our understanding of biodiversity in the Puget Sound region and has important implications for conservation. The findings show that eDNA samples collected from benthic regions yield higher species counts compared to surface samples and more accurately reflect where species were detected.

I have one major reservation about this study. Although the authors acknowledge that hybridization occurs within this genus, they do not address the limitations of detecting hybrids through eDNA sampling. This is particularly important when using mitochondrial loci, which only provide information on the maternal lineage. While the authors cite Schwenke et al. (2018) on page 3—where it is suggested that hybridization may be occurring in Puget Sound—this point is not further discussed in the manuscript.

I strongly recommend adding a paragraph in the discussion and conclusion sections to address the caveats of using eDNA, including the potential for underestimating species richness. Additionally, please include citations that discuss the limitations of eDNA and how integrating nuclear DNA data can help resolve hybridization issues.

We have amended the discussion to include the fact that mitochondrial assays cannot detect hybridization and added a sentence pointing out the potential of nuclear eDNA assays to investigate hybrids. However, both nuclear eDNA assays and species hybridization are beyond the scope of this manuscript, so we have kept this section brief.

Another helpful addition would be a flowchart illustrating the database development process. This could be included as supplemental information to clarify how primers were generated and applied.

We find that the written description of the database development is clearer than an infographic of flowchart, which would require significant amounts of text explaining the graphics. We have opted not to add this supplemental figure, and believe that readers interested in database development will find the described methods sufficient for understanding the process.

Below are minor comments to further improve the manuscript.

Line 52: china should be changed to China. Since the species name is the name of a country as well.

Change has been made.

Line 129: What are the version numbers for all bioinformatics programs (i.e. cutadapt, bowtie, phyloseq, etc)?

All version numbers have been added.

Line 231: There was not much information about data analysis scripts completed on the GitHub site.

We have added a description to the GitHub repository describing the data analysis scripts. All bioinformatic analysis code is included on this site.

Line 246: For consistency, remove spaces from S. hubbsi / S. longispinis to match other species pairs.

Change made, thank you.

Reviewer #2: The work by Matthews and collaborators reports the development of a metabarcoding assay to discriminate most of the rockfish species (genus Sebastes).
This is a very speciose genus, with many of its species inhabitting the same waters. Correctly detecting the presence of a species is very difficult, and doing so, regardless of the methods, requires a lot of effort.

The authors target the Dloop zone, and design a new set of primers to amplify a ~350 bp fragment able to discriminate almost all Sebastes species, and all of those expected in the Puget Sound (PS) and Hood Canal (HC). To do so, they increase the coverage of the Sebastes spp in public databases, by sequencing their genomes and assembling mitogenoms with genome skimming.

The authors design the primers and with the amplicons generate a classification tree using the R package insect. Then they test the assay with eDNA samples from HC and PS, detecting 7 rockfish species.

What are the standards needed to clear when designing a new assay? The authors use a set of them:
-Primer binding: One template tests for the species expected in HC and PS. Reporting positive results, unclear if successful amplicons were sequenced to confirm identity.
-Discrimination in silico
-Working with real samples

We have added clarification to the methods section of the manuscript, describing the sequencing and alignment of all amplicons for confirmation of species identity and target region amplification.

The one thing I am missing is a mock community assay, in which you test whether the relative performance of the primers in different taxa might deem some species undetactable, given that after 35 PCR cycles the relative proportion of amplicons from species with lower afinity might be too small to be detected.

This could help explain the low proportion of rockfish species detected (7 out of the 28 present, in 12 sites) - but maybe they are rare species and thus harder to detect.

Another possibility is that the bioinformatics pipeline, particularly at the LULU stage, is merging true ASVs into one OTU.
At the time of reviewing we don't have the ASV data available, nor the classification tree, so I can't

The reviewer brings up valid potential explanations for the low number of rockfish species detected relative to the total number of species with observation records in Puget Sound. However, the species we detected include all of the commonly observed species in the region. Many of the 28 species with detection records in the region have been observed only a few times, or have significant interannual variability and are unlikely to be encountered within a 2-3 day field campaign, by any method. We have expanded our discussion of the relative abundances and likelihoods of encounter for different species, and added a supplemental table ranking the probability of encounter, summarized from literature as well as expert experience.

Besides that, the article is well reasoned and written, with a few inline comments:

line 35: "state and federal governments" These are government bodies in the United States - rephrase to make it applicable to other countries (eg goverment bodies, managing agencies)

Change made, thank you.

line 36-37: The sentence sounds incomplete - please elaborate further.
We have rewritten this sentence and added clarification.

line 43 : Is species flock an accepted term?

Species flocks are groups of closely related species, most commonly evolved in an isolated region to fill different ecological roles. We find it appropriate to use here given the shared bathymetric and benthopelagic habitat of rockfishes, despite their broad geographic ranges.

Sentence from line 70 to 73. They way it is written seems to point at the large number of copies and database coverage as the reason these hypervariable regions are mitochondrial. I think the reason is that the mitochondrial DNA evolves at a higher rate than nuclear DNA([https://doi.org/10.1093/molbev/msx197](https://urldefense.com/v3/__https:/doi.org/10.1093/molbev/msx197__;!!K-Hz7m0Vt54!nQb11Tk8pmTbbMs8aJ-9W1RfmYAuoFVbbTqlyheztpgXiTiHwcKcCaqP3uVsuFyN_ampfDza_O37yA$)), and the control region does it at an even higher rate. Plus the heredability of the mitochondrial genome stops recombination and substitutions are the likely pattern of evolution.

We have rewritten this sentence to clarify that the hypervariable regions themselves are not necessarily more likely to be mitochondrial, but that eDNA research finds mitochondrial hypervariable regions more useful.

Line 82: probably another good reason is the length limitiation of the high quality NGS platforms.

We opted not to include this detail, given the advent of longer reads from the new Illumina MiSeq platform, as well as long-read capabilities of emerging platforms (e.g., Oxford Nanopore). We prefer to highlight the utility of the marker in a platform agnostic manner.

line 126: Typo in sequencing

Fixed, thank you.

line 128: You need to estate the trimming paremeters - particularly with the Qscore binning in NovaSeq data

Details for the sequence trimming have been added

line 129: You used cutadapt but no adapter trimming was made, correct?

We have added details describing how cutadapt was used.

line 160 : ...in situ and at depth, an Ascension eDNA sampler...

Change made.

FIGURE 1: The figure is indeed a map, but a very hard one to read. Can you re do the figure with easier color selections (one color for all terrain above water, maybe a window to the location of the sampling area with respect to North America's West Coast, remove "Downtown") so it is easier to read?

We thank the reviewer for these suggestions. We have edited the figure to include an inset showing the sampling area with respect to the west coast of North America, as well as to reduce the number of colors.

line 186: Spell out No template Control

Change made

line 190: We know there are 8 species pairs sharing reference sequences, but we don't know the minimum number of differences between species. Wouldn't it be better to find a suitable HiFi Taq for the whole process?

We acknowledge that a HiFi Taq may reduce error rates (but see Shaffer et al. 2025 for some investigation of the effects of different Taqs on species proportions: <https://onlinelibrary.wiley.com/doi/full/10.1111/1755-0998.14119>). However, we had success with the more cost-effective Hot-Start Platinum II Taq and this is what was used for our study. Our bioinformatic processing includes stringent quality filtering, including chimera detection in *dada2* and independent *Lulu* filtering to remove potential daughter OTUs arising from PCR and sequencing error.

line 218: Lulu's documentation as per the reference provided needs a match table between all detected OTUs - did you do that or does it work differently in a newer package?. I would prefer the ASV table and sequences to be shared, after and before Lulu.

We ran LULU as described in the cited reference, including generating a match table between all detected ASVs and subsequently filtering potential daughter ASVs (with minimum ratio of 1, match of 84, and co-occurrence of 0.95, as described in the manuscript).

We have included all scripts to re-generate these analyses from the raw fastq files, which are available through NCBI. We prefer not to provide a stand-alone version of the intermediate, non-LULU filtered ASV table, as the goal of this manuscript is not to validate the use of LULU. For the casual reader the unfiltered table is not useful for per-species comparisons as ASVs were not assigned taxonomic classification until after LULU filtering. For those interested in in-depth re-analysis of the data, the raw data and all bioinformatic scripts are available.

line 221: Need to provide the AIC thresold you allowed insect to work with. I assume most of the assignments had a score of NA (as the query sequence was already present in the database), but if there are many low values, maybe you are allowing species-level asignments when the algorithm is not too confident. report the cutoff value to get a sense of this. Computing the classification tree requires a lot of time and computer power. Are you sharing the tree as an .rds file?

We have amended the text to include both the minimum AIC threshold allowed and the minimum observed.

The .rds file with the trained classification tree is available on the linked Github, together with all bioinformatic scripts used to analyze the data. We have added the classification tree to the description of the data available on Github, to facilitate the reader finding it.

FIGURE 2: NJ tree is ok to reflect sequence similiarity, as it groups similar sequences first, and the phylogenetic relationship amongst all Sebastes species are beyond the scope of the paper. Despite that, both the MAFFT aligment and NJ tree specifications deserve to be in the method section.

Thank you for this suggestion. We have added a description of the methods used to generate the tree to the database development section of the methods.

line 330: I agree that the region differentiaties the deposited sequences, but only 7 species have been amplified and detected. One very possible explanation is the local absence of the species that did not show up, but another one could be that your primers do not bind very well. I know in table S1 you show the species by species tests, but what about if species are co-ocurring in a sample? Relative affinity to the primers could make the signal from the less favored species dissapear. You show that there are only between 0 and 3 mismatches between primers and deposited sequences, so maybe these differences do not account for much. I do not have enough evidence to go one way or the other, and only a mock community test can solve this. Same for the conclusion in lines 413-415.

We acknowledge that the detection of only 7 out of 28 species seems concerning. However, most of the 28 rockfish species with recorded observations in Puget Sound are rarely seen, and many are associated with habitats that we did not sample. We have added this information to the manuscript, as well as a summary ranking of likelihood of encounter based on expert opinion (now Table S6).

We also agree that mismatches have the potential to significantly bias relative abundances, even to the point of species with low amplification efficiency dropping out. However, the relatively low number of mismatches between our primers and target template make this unlikely to be a significant issue, as severe biases more typically occur when mismatches are greater than 6bp (Shaffer et al., 2025). We have now addressed this concern in the discussion, and thank the reviewer for bringing it up.

line 351: 5 of which are relatively to very common. Spell out Five, also in line 352

Change made

Regarding your data sharing and availability, please clarify that you'll be sharing:
-raw sequence data from genome skimming
-raw sequence data from metabarcoding MiSeq runs
-Insect classification tree used to identify the sequences generated
-ASV tables and sequences before and after Lulu

Raw and assmelbed data from genome skimming are available on NCBI. Raw MiSeq data from metabarcoding are available on NCBI. The classification tree and processed ASV table are available on Github, as described in the methods.
