# Supplementary material for: eDNA reveals spatial differences in species composition of protected rockfishes
Source: PLoS One. 2025 Dec 1;20(12):e0337724. doi: 10.1371/journal.pone.0337724 (PMC12668609; doi:10.1371/journal.pone.0337724)
Supplement: Attachment — Submitted filename: Response_to_Reviewers_10-28.docx [file pone.0337724.s006.docx]

Dear Dr. Fujiwara,

We thank the anonymous reviewer providing additional feedback, and yourself for comparing the revised manuscript against the second reviewer’s suggestions. We have incorporated the reviewer suggestions in the latest version of the manuscript. Please find below a point-by-point response to the suggested edits (Reviewer comments in black, author responses in red).

We have also submitted a PLOS Authorship Change Form as well as a letter signed by all coauthors requesting the change in authorship. The addition of Abigail Wells to the author list reflects her significant contributions in the form of initial primer development and conceptualization of the database. She has reviewed and edited the final version of the manuscript and agrees to be accountable for the work. She was not included as a coauthor in the original submission only because we were not able to get in touch with her prior to the original submission.

All the best,

Stephanie Matthews

PONE-D-25-30427R1

eDNA reveals spatial differences in species composition of protected rockfishes

PLOS ONE

Dear Dr. Matthews,

Thank you for submitting your manuscript to PLOS ONE. After careful consideration, we feel that it has merit but does not fully meet PLOS ONE’s publication criteria as it currently stands. Therefore, we invite you to submit a revised version of the manuscript that addresses the points raised during the review process.

We received feedback from one of the reviewers. Unfortunately, the comments from the other reviewer were not submitted in time. However, I carefully compared the revised manuscript with the original version and considered the comments provided by the second reviewer. I am satisfied that the major concerns have been fully addressed in the Discussion section by clearly outlining potential weaknesses and alternative interpretations. The other reviewer has suggested only minor revisions, which I believe can be addressed with relative ease.

I noticed a change in authorship, but I could not find any accompanying justification. Please include a brief explanation for this change in the cover letter upon resubmission.

Please submit your revised manuscript by Dec 11 2025 11:59PM. If you will need more time than this to complete your revisions, please reply to this message or contact the journal office at [plosone@plos.org](mailto:plosone@plos.org). When you're ready to submit your revision, log on to [https://www.editorialmanager.com/pone/](https://urldefense.com/v3/__https:/www.editorialmanager.com/pone/__;!!K-Hz7m0Vt54!mAUANSASg0xvdVJKbayrvlOcF75WeldCDVGpIogzitjQFw1E4eorzcg6ejk_DZlWra6UQbwDDHFTNQ$) and select the 'Submissions Needing Revision' folder to locate your manuscript file.

Please include the following items when submitting your revised manuscript:

- A rebuttal letter that responds to each point raised by the academic editor and reviewer(s). You should upload this letter as a separate file labeled 'Response to Reviewers'.
- A marked-up copy of your manuscript that highlights changes made to the original version. You should upload this as a separate file labeled 'Revised Manuscript with Track Changes'.
- An unmarked version of your revised paper without tracked changes. You should upload this as a separate file labeled 'Manuscript'.

If you would like to make changes to your financial disclosure, please include your updated statement in your cover letter. Guidelines for resubmitting your figure files are available below the reviewer comments at the end of this letter.

If applicable, we recommend that you deposit your laboratory protocols in [protocols.io](http://protocols.io/) to enhance the reproducibility of your results. Protocols.io assigns your protocol its own identifier (DOI) so that it can be cited independently in the future. For instructions see: [https://journals.plos.org/plosone/s/submission-guidelines#loc-laboratory-protocols](https://urldefense.com/v3/__https:/track.editorialmanager.com/CL0/https:*2F*2Fjournals.plos.org*2Fplosone*2Fs*2Fsubmission-guidelines*23loc-laboratory-protocols/1/010f019a284b90c5-2a2063dd-34e8-4f17-a473-09919b279850-000000/fK7cV_76yS88x5XrmF6JQBXtAdWPR19LNBwU5KNj7ZY=234__;JSUlJSUl!!K-Hz7m0Vt54!mAUANSASg0xvdVJKbayrvlOcF75WeldCDVGpIogzitjQFw1E4eorzcg6ejk_DZlWra6UQbx9NwN5ww$). Additionally, PLOS ONE offers an option for publishing peer-reviewed Lab Protocol articles, which describe protocols hosted on [protocols.io](http://protocols.io/). Read more information on sharing protocols at [https://plos.org/protocols?utm_medium=editorial-email&utm_source=authorletters&utm_campaign=protocols](https://urldefense.com/v3/__https:/track.editorialmanager.com/CL0/https:*2F*2Fplos.org*2Fprotocols*3Futm_medium=editorial-email*26utm_source=authorletters*26utm_campaign=protocols/1/010f019a284b90c5-2a2063dd-34e8-4f17-a473-09919b279850-000000/vNr1x5rjRor9GxN_qu-7qbcZnmlB3hp9P2Z2UE0b3p0=234__;JSUlJSUl!!K-Hz7m0Vt54!mAUANSASg0xvdVJKbayrvlOcF75WeldCDVGpIogzitjQFw1E4eorzcg6ejk_DZlWra6UQby5gNGEXA$).

We look forward to receiving your revised manuscript.

Kind regards,

Masami Fujiwara, PhD
Academic Editor
PLOS ONE

Journal Requirements:

If the reviewer comments include a recommendation to cite specific previously published works, please review and evaluate these publications to determine whether they are relevant and should be cited. There is no requirement to cite these works unless the editor has indicated otherwise. 

Please review your reference list to ensure that it is complete and correct. If you have cited papers that have been retracted, please include the rationale for doing so in the manuscript text, or remove these references and replace them with relevant current references. Any changes to the reference list should be mentioned in the rebuttal letter that accompanies your revised manuscript. If you need to cite a retracted article, indicate the article’s retracted status in the References list and also include a citation and full reference for the retraction notice.


[Note: HTML markup is below. Please do not edit.]

Reviewers' comments:

Reviewer's Responses to Questions

Reviewer #1: This study by Matthews et al. provides a valuable resource for monitoring the presence or absence of multiple rockfish species using eDNA. All of my major concerns and suggestions were satisfactorily addressed in the revised version of the manuscript, particularly those regarding the limitations of mitochondrial markers for eDNA applications. However, I believe several minor edits should still be addressed before publication. My specific comments and suggestions are listed below.
General comments
The resolution of the figures is low; please provide higher-resolution versions for publication quality.

We have uploaded TIFF files. Upon editorial request or acceptance, we can provide figures in another format if required.

There is inconsistency in citation formatting—some references are numbered, while others use author–year style. Please ensure the citation format is consistent throughout.

Changes made throughout.

Line-by-line comments:
Line 18: Remove italics from the closing parenthesis “)”.

Change made. (Now Line 18).

Line 51: Correct the spelling of bocaccio.

Change made. (Now Line 51).

Line 62: Spell out ROV (Remotely Operated Vehicle) and AUV (Autonomous Underwater Vehicle), as not all readers may be familiar with these abbreviations.

Change made. (Now Line 62-63).

Line 101: The D-loop primers from Hyde and Vetter (2007) are mentioned, but their positions relative to the primers developed in this study are unclear. Please clarify this comparison.

We have added a description of the binding locations for the Hyde & Vetter (2007) primers, as well as the binding locations of the newly described primers relative to the Hyde and Vetter (2007) primers. The text now reads:

The Hyde and Vetter primers bind in the 3’ region of the cytochrome b gene and approximately ~370 bp from the 5’ end of the D-loop region, amplifying the tRNA-Thr and tRNA-Pro genes as well as the first ~370 bp of the D-loop. However, the amplicons generated by these primers are ~535 bp and outside the length range that returns high quality data for amplicon sequencing on the Illumina MiSeq. Using publicly available sequence data combined with preliminary sequence data from pilot eDNA samples, we designed a set of primers internal to the Hyde and Vetter primers, spanning approximately 350 bp. The newly designed forward primer is located 132 bp downstream of the Hyde and Vetter forward primer, within the tRNA-Pro gene; the reverse primer is located 83 bp upstream of the Hyde and Vetter reverse primer, within the D-loop.

(Now Lines 100-109).

Line 105: Provide the name of the exceptional rockfish species mentioned here.

We have added the species name (*S. entomelas*), and explained that this is because the tissue sample of *S. entomelas* that we obtained was instead from *S. flavidus.* (Now Lines 111-112).

Line 169: Use the full genus name (Sebastolobus alascanus) since this is the first mention of the species.

No change made, as the full species name *Sebastolobus alascanus* already appears earlier in this paragraph. (Now Line 173).

Line 190: Spell out CTD (Conductivity, Temperature, Depth).

Change made. (Now Line 197).

Line 284: Sebastes should be italicized.

Change made. (Now Line 292).

Line 296: Include examples of fishes in the family Scombridae (e.g., tunas, mackerel, and bonito).

Change made. (Now lines 303-304).

Lines 371–372: Ensure consistency in listing species names and their common names. When a species is first mentioned, include its common name.

We have updated the text to always refer primarily to species name, but to include the common name upon first mention within a section. Text also updated at lines 173, 285-289 (Table 1), 323-329, 417.

Line 396: Consider referring to Figure 1 here, as Admiralty Inlet and Hood Canal are discussed in this section.

Change made. (Now lines 405-406).

Line 458: Insert a space between “184” and “bp.”

Change made. (Now line 467)

Figure 2 legend: Include a note indicating that the scale bar represents genetic distance (e.g., “Scale bar = 0.02 genetic distance”).

Description added. We also noted that the version of the figure included in the manuscript uses a different method than previously reported (UPGMA rather than neighbor-joining, using the same alignment as originally reported). We have updated the methods and legend for accuracy. (Now line 296, also updated at 176-177)
